# *N*-(2,6-Dimethyl­phen­yl)benzamide

**DOI:** 10.1107/S1600536808018230

**Published:** 2008-06-19

**Authors:** B. Thimme Gowda, Miroslav Tokarčík, Jozef Kožíšek, B. P. Sowmya, Hartmut Fuess

**Affiliations:** a Department of Chemistry, Mangalore University, Mangalagangotri 574 199, Mangalore, India; b Faculty of Chemical and Food Technology, Slovak Technical University, Radlinského 9, SK-812 37 Bratislava, Slovak Republic; c Institute of Materials Science, Darmstadt University of Technology, Petersenstrasse 23, D-64287 Darmstadt, Germany

## Abstract

The title compound, C_15_H_15_NO, crystallizes with two mol­ecules in the asymmetric unit. The H—N—C=O units are in a *trans* conformation, similar to that observed in *N*-(3,4-dimethyl­phen­yl)benzamide, *N*-(2,6-dichloro­phen­yl)benz­amide and other benzanilides. The central –NHCO– bridging unit is tilted at angles of 17.1 (3) and 16.4 (3)° to the benzoyl ring in the two mol­ecules. The two rings (benzoyl and aniline) are almost orthogonal with respect to each other, making dihedral angles of 86.3 (1) and 86.0 (1)° in the two mol­ecules. N—H⋯O hydrogen bonds link mol­ecules into infinite chains running along the *c* axis.

## Related literature

For related literature, see: Gowda *et al.* (2003 ▶, 2008*a* ▶,*b* ▶).
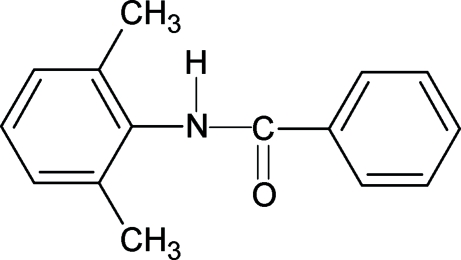


## Experimental

### 

#### Crystal data

C_15_H_15_NO
*M* *_r_* = 225.28
Monoclinic, 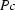

*a* = 16.4389 (8) Å
*b* = 8.2903 (4) Å
*c* = 9.4902 (3) Å
β = 98.165 (4)°
*V* = 1280.25 (10) Å^3^
*Z* = 4
Mo *K*α radiation
μ = 0.07 mm^−1^
*T* = 295 (2) K
0.52 × 0.46 × 0.22 mm

#### Data collection

Oxford Diffraction Xcalibur diffractometer
Absorption correction: multi-scan (*CrysAlis RED*; Oxford Diffraction, 2007 ▶) *T*_min_ = 0.924, *T*_max_ = 0.985
38015 measured reflections
2504 independent reflections
2110 reflections with *I* > 2σ(*I*)
*R*_int_ = 0.059

#### Refinement

*R*[*F* ^2^ > 2σ(*F* ^2^)] = 0.039
*wR*(*F* ^2^) = 0.108
*S* = 0.98
2504 reflections
307 parameters
35 restraints
H-atom parameters constrained
Δρ_max_ = 0.13 e Å^−3^
Δρ_min_ = −0.14 e Å^−3^

### 

Data collection: *CrysAlis CCD* (Oxford Diffraction, 2007 ▶); cell refinement: *CrysAlis RED* (Oxford Diffraction, 2007 ▶); data reduction: *CrysAlis RED*; program(s) used to solve structure: *SHELXS97* (Sheldrick, 2008 ▶); program(s) used to refine structure: *SHELXL97* (Sheldrick, 2008 ▶); molecular graphics: *ORTEP-3* (Farrugia, 1997 ▶) and *DIAMOND* (Brandenburg, 2002 ▶); software used to prepare material for publication: *SHELXL97*, *PLATON* (Spek, 2003 ▶) and *WinGX* (Farrugia, 1999 ▶).

## Supplementary Material

Crystal structure: contains datablocks I, global. DOI: 10.1107/S1600536808018230/bt2724sup1.cif

Structure factors: contains datablocks I. DOI: 10.1107/S1600536808018230/bt2724Isup2.hkl

Additional supplementary materials:  crystallographic information; 3D view; checkCIF report

## Figures and Tables

**Table 1:** Hydrogen-bond geometry (Å, °)

*D*—H⋯*A*	*D*—H	H⋯*A*	*D*⋯*A*	*D*—H⋯*A*
N1—H1N⋯O1^i^	0.86	2.19	2.949 (2)	147
N2—H2N⋯O2^ii^	0.86	2.17	2.949 (2)	150

Symmetry codes: (i) 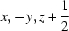; (ii) 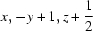.

**Fig. 1.**
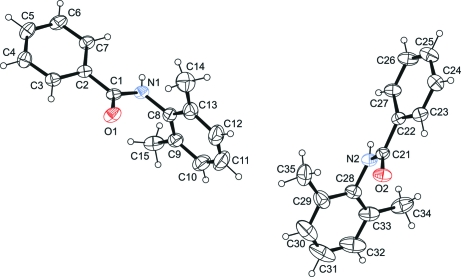
Molecular structure of the title compound showing the atom labelling scheme. Displacement ellipsoids are drawn at the 30% probability level. H atoms are represented as small spheres of arbitrary radii.

**Fig. 2.**
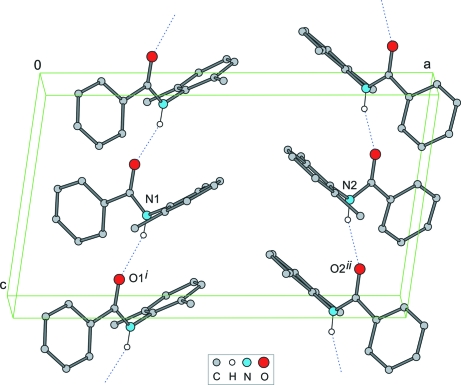
Crystal structure of the title compound viewed down the b axis. Hydrogen bonds N1–H1N···O1(i) and N2–H2N···O2(ii) give rise to infinite molecular chains running along the c axis. Symmetry codes: (i) x,-y,z + 1/2; (ii) x,-y + 1,z + 1/2. H atoms not involved in hydrogen bonding are omitted.

## supplementary crystallographic information

### Comment

In the present work, the structure of *N*-(2,6-dimethylphenyl)-benzamide (N26DMPBA) has been determined to study the effect of substituents on the solid state geometries of benzanilides (Gowda *et al.*, 2003; Gowda *et al.*, 2008a; Gowda *et al.*, 2008b). The conformations of the N—H and C=O bonds in N26DMPBA (Fig.1) are anti to each other, similar to that observed in *N*-(3,4-dimethylphenyl)-benzamide (Gowda *et al.*, 2008*a*), *N*-(2,6-dichlorophenyl)-benzamide (Gowda *et al.*, 2008*b*) and other benzanilides (Gowda *et al.*, 2003). The structure of N26DMPBA has two molecules in its asymmetric unit. The central amide group –NHCO– is tilted to the benzoyl ring at the angles of 17.1 (3)° and 16.4 (3)°, in molecule 1 and 2, respectively. The two rings (benzoyl and aniline) are almost orthogonal, with the dihedral angles of 86.3 (1)° and 86.0 (1)° in molecules 1 and 2, respectively.

Part of the crystal structure of the title compound showing molecular chains as viewed down the *b* axis is shown in Fig.2. Hydrogen bonds N1–H1N···O1(i) and N2–H2N···O2(ii) give rise to infinite molecular chains running along the *c* axis (Symmetry codes: (i) *x*,-*y*,*z* + 1/2; (ii) *x*,-*y* + 1,*z* + 1/2).

### Experimental

The title compound was prepared according to the literature method (Gowda *et al.*, 2003). The purity of the compound was checked by determining its melting point. It was characterized by recording its infrared and NMR spectra. Single crystals of the title compound were obtained from an ethanolic solution and used for X-ray diffraction studies at room temperature.

### Refinement

All H atoms were placed in calculated positions and constrained to ride on their parent atoms, with C–H distances of 0.93Å (C-aromatic), 0.96Å (*C*-methyl) and N–H distances 0.86 Å. The *U*_iso_(H) values were set at 1.2 *U*_eq_(C,*N*) and 1.5 *U*_eq_ (*C*-methyl). The displacement parameters of C-atoms in aniline ring of both molecules were restrained by use of the *SHELXL97* DELU command with default standard deviations.

### Crystal data

**Table:**

C_15_H_15_NO	*F*_000_ = 480
*M**_r_* = 225.28	*D*_x_ = 1.169 Mg m^−^^3^
Monoclinic, *P**c*	Mo *K*α radiation λ = 0.71073 Å
Hall symbol: P -2 y c	Cell parameters from 18913 reflections
*a* = 16.4389 (8) Å	θ = 3.1–29.3º
*b* = 8.2903 (4) Å	µ = 0.07 mm^−^^1^
*c* = 9.4902 (3) Å	*T* = 295 (2) K
β = 98.165 (4)º	Block, colourless
*V* = 1280.25 (10) Å^3^	0.52 × 0.46 × 0.22 mm
*Z* = 4	

### Data collection

**Table:**

Oxford Diffraction Xcalibur diffractometer	2504 independent reflections
Monochromator: graphite	2110 reflections with *I* > 2σ(*I*)
Detector resolution: 10.434 pixels mm^-1^	*R*_int_ = 0.059
*T* = 295(2) K	θ_max_ = 26.0º
ω scans with κ offsets	θ_min_ = 5.3º
Absorption correction: multi-scan(CrysAlis RED; Oxford Diffraction, 2007)	*h* = −20→20
*T*_min_ = 0.924, *T*_max_ = 0.985	*k* = −10→10
38015 measured reflections	*l* = −11→11

### Refinement

**Table:**

Refinement on *F*^2^	Secondary atom site location: difference Fourier map
Least-squares matrix: full	Hydrogen site location: inferred from neighbouring sites
*R*[*F*^2^ > 2σ(*F*^2^)] = 0.039	H-atom parameters constrained
*wR*(*F*^2^) = 0.108	*w* = 1/[σ^2^(*F*_o_^2^) + (0.0851*P*)^2^] where *P* = (*F*_o_^2^ + 2*F*_c_^2^)/3
*S* = 0.98	(Δ/σ)_max_ = 0.001
2504 reflections	Δρ_max_ = 0.13 e Å^−^^3^
307 parameters	Δρ_min_ = −0.14 e Å^−^^3^
35 restraints	Extinction correction: none
Primary atom site location: structure-invariant direct methods	Absolute structure: Flack (1983), 2493 Friedel pairs

### Special details

**Table:**

Geometry. All e.s.d.'s (except the e.s.d. in the dihedral angle between two l.s. planes) are estimated using the full covariance matrix. The cell e.s.d.'s are taken into account individually in the estimation of e.s.d.'s in distances, angles and torsion angles; correlations between e.s.d.'s in cell parameters are only used when they are defined by crystal symmetry. An approximate (isotropic) treatment of cell e.s.d.'s is used for estimating e.s.d.'s involving l.s. planes.
Refinement. Refinement of *F*^2^ against ALL reflections. The weighted *R*-factor *wR* and goodness of fit *S* are based on *F*^2^, conventional *R*-factors *R* are based on *F*, with *F* set to zero for negative *F*^2^. The threshold expression of *F*^2^ > σ(*F*^2^) is used only for calculating *R*-factors(gt) *etc*. and is not relevant to the choice of reflections for refinement. *R*-factors based on *F*^2^ are statistically about twice as large as those based on *F*, and *R*- factors based on ALL data will be even larger.

### Fractional atomic coordinates and isotropic or equivalent isotropic displacement parameters (Å^2^)

**Table:**

	*x*	*y*	*z*	*U*_iso_*/*U*_eq_	
N1	0.32493 (12)	0.0027 (2)	0.63912 (17)	0.0476 (4)	
H1N	0.324	−0.0067	0.7291	0.057*	
O1	0.27687 (12)	−0.0838 (2)	0.41847 (15)	0.0654 (5)	
C1	0.27534 (13)	−0.0900 (2)	0.5470 (2)	0.0446 (5)	
C2	0.21752 (13)	−0.2015 (2)	0.6052 (2)	0.0435 (5)	
C3	0.15307 (16)	−0.2637 (3)	0.5109 (2)	0.0523 (5)	
H3	0.1478	−0.2349	0.4153	0.063*	
C4	0.09687 (16)	−0.3669 (3)	0.5561 (3)	0.0601 (6)	
H4	0.0532	−0.4056	0.492	0.072*	
C5	0.10537 (18)	−0.4130 (3)	0.6967 (3)	0.0664 (7)	
H5	0.0679	−0.4843	0.7276	0.08*	
C6	0.16901 (17)	−0.3537 (3)	0.7909 (3)	0.0655 (7)	
H6	0.1745	−0.3852	0.8859	0.079*	
C7	0.22549 (17)	−0.2472 (3)	0.7468 (2)	0.0534 (5)	
H7	0.2683	−0.2068	0.8118	0.064*	
C8	0.37898 (15)	0.1165 (3)	0.5877 (2)	0.0523 (5)	
C9	0.36263 (18)	0.2796 (3)	0.5956 (3)	0.0611 (6)	
C10	0.4128 (2)	0.3869 (4)	0.5370 (4)	0.0872 (10)	
H10	0.4028	0.4971	0.5414	0.105*	
C11	0.4773 (3)	0.3323 (6)	0.4721 (5)	0.1051 (13)	
H11	0.5097	0.4056	0.431	0.126*	
C12	0.4940 (2)	0.1719 (6)	0.4678 (4)	0.0922 (10)	
H12	0.5382	0.1373	0.4245	0.111*	
C13	0.44632 (17)	0.0583 (4)	0.5268 (3)	0.0714 (7)	
C14	0.46683 (19)	−0.1163 (3)	0.5241 (4)	0.0916 (10)	
H14A	0.5245	−0.1288	0.5198	0.137*	
H14B	0.4358	−0.1657	0.4421	0.137*	
H14C	0.4535	−0.1671	0.6088	0.137*	
C15	0.29134 (19)	0.3401 (3)	0.6634 (3)	0.0813 (8)	
H15A	0.3035	0.329	0.7649	0.122*	
H15B	0.2817	0.4516	0.6394	0.122*	
H15C	0.2432	0.2782	0.6291	0.122*	
N2	0.82433 (12)	0.5011 (2)	0.52006 (18)	0.0491 (4)	
H2N	0.8272	0.4924	0.6109	0.059*	
O2	0.87411 (12)	0.4211 (2)	0.32326 (16)	0.0615 (5)	
C21	0.87462 (13)	0.4123 (3)	0.4518 (2)	0.0432 (5)	
C22	0.93234 (13)	0.2984 (2)	0.5387 (2)	0.0432 (5)	
C23	0.99704 (15)	0.2371 (3)	0.4762 (2)	0.0524 (5)	
H23	1.0031	0.268	0.3841	0.063*	
C24	1.05223 (16)	0.1314 (3)	0.5484 (3)	0.0617 (6)	
H24	1.0959	0.0927	0.5058	0.074*	
C25	1.04286 (19)	0.0830 (4)	0.6833 (3)	0.0695 (7)	
H25	1.0798	0.0102	0.7319	0.083*	
C26	0.9794 (2)	0.1416 (4)	0.7462 (3)	0.0706 (7)	
H26	0.9733	0.1089	0.8379	0.085*	
C27	0.92362 (17)	0.2497 (3)	0.6748 (2)	0.0552 (6)	
H27	0.8805	0.2892	0.7185	0.066*	
C28	0.76616 (16)	0.6096 (3)	0.4456 (3)	0.0574 (6)	
C29	0.69959 (18)	0.5439 (5)	0.3541 (3)	0.0782 (8)	
C30	0.6445 (3)	0.6494 (8)	0.2804 (4)	0.1161 (14)	
H30	0.5997	0.6093	0.2198	0.139*	
C31	0.6549 (4)	0.8115 (8)	0.2950 (5)	0.1338 (18)	
H31	0.6174	0.8807	0.2432	0.161*	
C32	0.7200 (3)	0.8750 (5)	0.3850 (5)	0.1149 (14)	
H32	0.7256	0.9862	0.3944	0.138*	
C33	0.7778 (2)	0.7734 (4)	0.4626 (3)	0.0749 (8)	
C34	0.8494 (3)	0.8415 (4)	0.5588 (4)	0.0956 (11)	
H34A	0.8985	0.7854	0.5439	0.143*	
H34B	0.8406	0.8288	0.6561	0.143*	
H34C	0.855	0.954	0.5383	0.143*	
C35	0.6868 (2)	0.3667 (5)	0.3381 (4)	0.0944 (11)	
H35A	0.6348	0.3462	0.2812	0.142*	
H35B	0.6876	0.3186	0.4303	0.142*	
H35C	0.73	0.3209	0.2924	0.142*	

### Atomic displacement parameters (Å^2^)

**Table:**

	*U*^11^	*U*^22^	*U*^33^	*U*^12^	*U*^13^	*U*^23^
N1	0.0636 (11)	0.0502 (11)	0.0299 (8)	−0.0042 (8)	0.0098 (7)	−0.0009 (7)
O1	0.0973 (13)	0.0703 (11)	0.0302 (8)	−0.0220 (9)	0.0143 (8)	−0.0006 (7)
C1	0.0622 (13)	0.0405 (11)	0.0317 (10)	0.0035 (9)	0.0088 (9)	0.0025 (8)
C2	0.0584 (12)	0.0382 (10)	0.0354 (10)	0.0056 (9)	0.0121 (9)	−0.0022 (8)
C3	0.0676 (14)	0.0524 (12)	0.0370 (10)	0.0002 (11)	0.0074 (10)	−0.0012 (9)
C4	0.0624 (14)	0.0608 (15)	0.0580 (14)	−0.0097 (12)	0.0113 (11)	−0.0109 (11)
C5	0.0785 (16)	0.0621 (15)	0.0651 (16)	−0.0169 (13)	0.0318 (13)	−0.0066 (12)
C6	0.0941 (19)	0.0658 (15)	0.0404 (12)	−0.0114 (14)	0.0224 (12)	0.0041 (11)
C7	0.0704 (15)	0.0549 (13)	0.0356 (10)	−0.0044 (11)	0.0096 (10)	0.0001 (9)
C8	0.0583 (12)	0.0603 (14)	0.0375 (11)	−0.0109 (10)	0.0040 (9)	0.0008 (9)
C9	0.0758 (16)	0.0583 (14)	0.0465 (13)	−0.0119 (12)	−0.0011 (11)	0.0019 (10)
C10	0.112 (2)	0.0695 (19)	0.077 (2)	−0.0283 (17)	0.0019 (18)	0.0093 (15)
C11	0.102 (3)	0.116 (3)	0.099 (3)	−0.047 (2)	0.021 (2)	0.018 (2)
C12	0.0732 (18)	0.118 (3)	0.089 (2)	−0.0255 (19)	0.0234 (17)	0.001 (2)
C13	0.0634 (15)	0.090 (2)	0.0615 (16)	−0.0082 (14)	0.0117 (13)	−0.0007 (14)
C14	0.083 (2)	0.097 (2)	0.100 (3)	0.0162 (18)	0.0284 (18)	−0.0079 (19)
C15	0.116 (2)	0.0602 (16)	0.0685 (17)	0.0098 (16)	0.0150 (16)	0.0055 (14)
N2	0.0646 (11)	0.0517 (11)	0.0318 (9)	0.0042 (9)	0.0095 (7)	0.0007 (7)
O2	0.0854 (11)	0.0690 (11)	0.0316 (8)	0.0136 (8)	0.0131 (7)	0.0056 (7)
C21	0.0534 (11)	0.0406 (11)	0.0354 (11)	−0.0037 (9)	0.0056 (9)	0.0006 (8)
C22	0.0566 (12)	0.0389 (10)	0.0337 (10)	−0.0058 (9)	0.0050 (9)	−0.0026 (8)
C23	0.0650 (14)	0.0505 (13)	0.0431 (11)	0.0003 (11)	0.0126 (10)	−0.0022 (9)
C24	0.0620 (14)	0.0627 (15)	0.0594 (14)	0.0096 (12)	0.0054 (11)	−0.0100 (11)
C25	0.0839 (18)	0.0638 (16)	0.0554 (15)	0.0191 (14)	−0.0085 (13)	−0.0017 (12)
C26	0.103 (2)	0.0713 (16)	0.0367 (12)	0.0173 (16)	0.0076 (12)	0.0101 (11)
C27	0.0738 (15)	0.0550 (13)	0.0378 (11)	0.0078 (11)	0.0109 (11)	0.0026 (10)
C28	0.0638 (14)	0.0676 (15)	0.0428 (12)	0.0141 (11)	0.0149 (10)	0.0050 (10)
C29	0.0598 (15)	0.117 (2)	0.0581 (16)	0.0097 (16)	0.0087 (12)	0.0036 (16)
C30	0.083 (2)	0.167 (4)	0.093 (3)	0.041 (3)	−0.006 (2)	0.006 (3)
C31	0.133 (4)	0.157 (4)	0.105 (3)	0.079 (3)	−0.006 (3)	0.026 (3)
C32	0.163 (4)	0.086 (2)	0.098 (3)	0.058 (3)	0.025 (3)	0.015 (2)
C33	0.106 (2)	0.0647 (17)	0.0579 (16)	0.0229 (15)	0.0245 (15)	0.0070 (12)
C34	0.155 (3)	0.0564 (17)	0.078 (2)	−0.009 (2)	0.024 (2)	−0.0030 (15)
C35	0.0733 (19)	0.114 (3)	0.094 (3)	−0.0303 (19)	0.0074 (18)	−0.014 (2)

### Geometric parameters (Å, °)

**Table:**

N1—C1	1.348 (3)	N2—C21	1.341 (3)
N1—C8	1.429 (3)	N2—C28	1.425 (3)
N1—H1N	0.86	N2—H2N	0.86
O1—C1	1.225 (2)	O2—C21	1.221 (3)
C1—C2	1.487 (3)	C21—C22	1.500 (3)
C2—C3	1.385 (3)	C22—C27	1.380 (3)
C2—C7	1.385 (3)	C22—C23	1.386 (3)
C3—C4	1.373 (4)	C23—C24	1.372 (4)
C3—H3	0.93	C23—H23	0.93
C4—C5	1.376 (4)	C24—C25	1.372 (4)
C4—H4	0.93	C24—H24	0.93
C5—C6	1.368 (4)	C25—C26	1.364 (4)
C5—H5	0.93	C25—H25	0.93
C6—C7	1.389 (4)	C26—C27	1.388 (4)
C6—H6	0.93	C26—H26	0.93
C7—H7	0.93	C27—H27	0.93
C8—C9	1.383 (4)	C28—C33	1.378 (4)
C8—C13	1.405 (4)	C28—C29	1.406 (4)
C9—C10	1.382 (4)	C29—C30	1.376 (5)
C9—C15	1.501 (4)	C29—C35	1.489 (6)
C10—C11	1.376 (6)	C30—C31	1.359 (8)
C10—H10	0.93	C30—H30	0.93
C11—C12	1.360 (6)	C31—C32	1.375 (8)
C11—H11	0.93	C31—H31	0.93
C12—C13	1.392 (5)	C32—C33	1.398 (5)
C12—H12	0.93	C32—H32	0.93
C13—C14	1.488 (4)	C33—C34	1.494 (5)
C14—H14A	0.96	C34—H34A	0.96
C14—H14B	0.96	C34—H34B	0.96
C14—H14C	0.96	C34—H34C	0.96
C15—H15A	0.96	C35—H35A	0.96
C15—H15B	0.96	C35—H35B	0.96
C15—H15C	0.9599	C35—H35C	0.96
			
C1—N1—C8	120.20 (17)	C21—N2—C28	121.55 (17)
C1—N1—H1N	119.9	C21—N2—H2N	119.2
C8—N1—H1N	119.9	C28—N2—H2N	119.2
O1—C1—N1	121.7 (2)	O2—C21—N2	122.2 (2)
O1—C1—C2	120.00 (19)	O2—C21—C22	120.06 (19)
N1—C1—C2	118.25 (17)	N2—C21—C22	117.74 (17)
C3—C2—C7	118.9 (2)	C27—C22—C23	118.8 (2)
C3—C2—C1	117.52 (18)	C27—C22—C21	123.8 (2)
C7—C2—C1	123.5 (2)	C23—C22—C21	117.35 (17)
C4—C3—C2	121.1 (2)	C24—C23—C22	120.9 (2)
C4—C3—H3	119.4	C24—C23—H23	119.6
C2—C3—H3	119.4	C22—C23—H23	119.6
C3—C4—C5	119.8 (2)	C23—C24—C25	120.0 (2)
C3—C4—H4	120.1	C23—C24—H24	120
C5—C4—H4	120.1	C25—C24—H24	120
C6—C5—C4	119.8 (2)	C26—C25—C24	119.9 (2)
C6—C5—H5	120.1	C26—C25—H25	120
C4—C5—H5	120.1	C24—C25—H25	120
C5—C6—C7	120.9 (2)	C25—C26—C27	120.7 (2)
C5—C6—H6	119.6	C25—C26—H26	119.7
C7—C6—H6	119.6	C27—C26—H26	119.7
C2—C7—C6	119.4 (2)	C22—C27—C26	119.8 (2)
C2—C7—H7	120.3	C22—C27—H27	120.1
C6—C7—H7	120.3	C26—C27—H27	120.1
C9—C8—C13	121.9 (2)	C33—C28—C29	122.5 (3)
C9—C8—N1	119.5 (2)	C33—C28—N2	119.5 (3)
C13—C8—N1	118.6 (2)	C29—C28—N2	118.1 (3)
C10—C9—C8	118.4 (3)	C30—C29—C28	117.8 (4)
C10—C9—C15	120.3 (3)	C30—C29—C35	120.2 (4)
C8—C9—C15	121.3 (2)	C28—C29—C35	122.1 (3)
C11—C10—C9	120.6 (3)	C31—C30—C29	120.8 (4)
C11—C10—H10	119.7	C31—C30—H30	119.6
C9—C10—H10	119.7	C29—C30—H30	119.6
C12—C11—C10	120.5 (3)	C30—C31—C32	121.1 (4)
C12—C11—H11	119.8	C30—C31—H31	119.4
C10—C11—H11	119.8	C32—C31—H31	119.4
C11—C12—C13	121.5 (3)	C31—C32—C33	120.5 (4)
C11—C12—H12	119.3	C31—C32—H32	119.8
C13—C12—H12	119.3	C33—C32—H32	119.8
C12—C13—C8	117.0 (3)	C28—C33—C32	117.3 (4)
C12—C13—C14	120.6 (3)	C28—C33—C34	121.9 (3)
C8—C13—C14	122.3 (2)	C32—C33—C34	120.8 (3)
C13—C14—H14A	109.5	C33—C34—H34A	109.5
C13—C14—H14B	109.5	C33—C34—H34B	109.5
H14A—C14—H14B	109.5	H34A—C34—H34B	109.5
C13—C14—H14C	109.5	C33—C34—H34C	109.5
H14A—C14—H14C	109.5	H34A—C34—H34C	109.5
H14B—C14—H14C	109.5	H34B—C34—H34C	109.5
C9—C15—H15A	109.5	C29—C35—H35A	109.5
C9—C15—H15B	109.5	C29—C35—H35B	109.5
H15A—C15—H15B	109.5	H35A—C35—H35B	109.5
C9—C15—H15C	109.4	C29—C35—H35C	109.5
H15A—C15—H15C	109.5	H35A—C35—H35C	109.5
H15B—C15—H15C	109.5	H35B—C35—H35C	109.5
			
C8—N1—C1—O1	2.5 (3)	C28—N2—C21—O2	1.0 (3)
C8—N1—C1—C2	−177.4 (2)	C28—N2—C21—C22	−178.5 (2)
O1—C1—C2—C3	−16.4 (3)	O2—C21—C22—C27	−162.8 (2)
N1—C1—C2—C3	163.44 (19)	N2—C21—C22—C27	16.7 (3)
O1—C1—C2—C7	162.8 (2)	O2—C21—C22—C23	15.9 (3)
N1—C1—C2—C7	−17.3 (3)	N2—C21—C22—C23	−164.55 (19)
C7—C2—C3—C4	1.0 (3)	C27—C22—C23—C24	−0.8 (3)
C1—C2—C3—C4	−179.8 (2)	C21—C22—C23—C24	−179.6 (2)
C2—C3—C4—C5	−1.5 (4)	C22—C23—C24—C25	1.2 (4)
C3—C4—C5—C6	1.0 (4)	C23—C24—C25—C26	−0.9 (4)
C4—C5—C6—C7	0.0 (4)	C24—C25—C26—C27	0.3 (5)
C3—C2—C7—C6	0.0 (3)	C23—C22—C27—C26	0.2 (3)
C1—C2—C7—C6	−179.2 (2)	C21—C22—C27—C26	178.9 (2)
C5—C6—C7—C2	−0.5 (4)	C25—C26—C27—C22	0.1 (4)
C1—N1—C8—C9	108.7 (2)	C21—N2—C28—C33	−110.2 (3)
C1—N1—C8—C13	−69.7 (3)	C21—N2—C28—C29	68.3 (3)
C13—C8—C9—C10	2.4 (4)	C33—C28—C29—C30	−0.3 (4)
N1—C8—C9—C10	−175.9 (2)	N2—C28—C29—C30	−178.8 (3)
C13—C8—C9—C15	−178.9 (3)	C33—C28—C29—C35	−179.0 (3)
N1—C8—C9—C15	2.8 (3)	N2—C28—C29—C35	2.5 (4)
C8—C9—C10—C11	0.1 (4)	C28—C29—C30—C31	0.6 (6)
C15—C9—C10—C11	−178.7 (3)	C35—C29—C30—C31	179.4 (4)
C9—C10—C11—C12	−1.6 (6)	C29—C30—C31—C32	−0.9 (8)
C10—C11—C12—C13	0.7 (6)	C30—C31—C32—C33	0.8 (8)
C11—C12—C13—C8	1.6 (5)	C29—C28—C33—C32	0.3 (4)
C11—C12—C13—C14	−178.5 (3)	N2—C28—C33—C32	178.7 (3)
C9—C8—C13—C12	−3.2 (4)	C29—C28—C33—C34	−179.2 (3)
N1—C8—C13—C12	175.1 (2)	N2—C28—C33—C34	−0.8 (4)
C9—C8—C13—C14	176.9 (3)	C31—C32—C33—C28	−0.5 (6)
N1—C8—C13—C14	−4.8 (4)	C31—C32—C33—C34	179.0 (4)

### Hydrogen-bond geometry (Å, °)

**Table:**

*D*—H···*A*	*D*—H	H···*A*	*D*···*A*	*D*—H···*A*
N1—H1N···O1^i^	0.86	2.19	2.949 (2)	147
N2—H2N···O2^ii^	0.86	2.17	2.949 (2)	150

Symmetry codes: (i) *x*, −*y*, *z*+1/2; (ii) *x*, −*y*+1, *z*+1/2.
